# Critical Care Ultrasonography for Cardiogenic Shock: A Scoping Review

**DOI:** 10.1097/CCE.0000000000001388

**Published:** 2026-03-12

**Authors:** Sameer Sharif, Kevin Wang, John Basmaji, Enyo Ablordeppey, José L. Díaz-Gómez, Michael Lanspa, Sara Nikravan, Kimberley Lewis

**Affiliations:** 1 Department of Medicine, Division of Emergency Medicine, McMaster University, Hamilton, ON, Canada.; 2 Department of Medicine, Division of Critical Care, McMaster University, Hamilton, ON, Canada.; 3 Department of Health Research Methods, Evidence and Impact, McMaster University, Hamilton, ON, Canada.; 4 McMaster University, Hamilton, ON, Canada.; 5 Western University, London, ON, Canada.; 6 Washington University, St. Louis, MO.; 7 Baylor College of Medicine, Houston, TX.; 8 Intermountain Medical Center, Murray, UT.; 9 University of Washington, Seattle, WA.

**Keywords:** cardiogenic shock, critical care ultrasonography, randomized, scoping review

## Abstract

**OBJECTIVES:**

To summarize the effectiveness of critical care ultrasonography (CCUS) in adult patients with cardiogenic shock vs. standard of care without CCUS on patient-relevant outcomes.

**DESIGN:**

We performed a scoping review across MEDLINE, Embase, CENTRAL, World Health Organization, International Clinical Trials Registry, ClinicalTrials.gov, and published and unpublished sources from inception until February 2024.

**SETTING:**

The emergency department, ward, or ICU.

**PATIENTS:**

We included randomized clinical trials (RCTs) and observational studies comparing CCUS to non-CCUS care in adult patients with cardiogenic shock. We included any type of ultrasound measure for the intervention in adult patients (≥ 18 yr old).

**INTERVENTIONS:**

CCUS.

**MEASUREMENTS AND MAIN RESULTS:**

We included two RCTs (*n* = 573 patients) and one observational study (*n* = 30 patients). RCT data suggested that CCUS, with transesophageal echocardiography in particular, in adult patients with cardiogenic shock may shorten time to resolution of hemodynamic instability at 72 hours (subhazard ratio [SHR], 1.26; 95% CI, 1.02–1.55) but failed to influence mortality (risk difference, –0.03; 95% CI, –0.1 to 0.05), time to resolution of hemodynamic instability within 6 days (SHR, 1.20; 95% CI, 0.98–1.46), ICU length of stay (LOS; *p* = 0.87), hospital LOS (*p* = 0.91), duration of mechanical ventilation (*p* = 0.73), or duration of renal replacement therapy (RRT; *p* = 0.68).

**CONCLUSIONS:**

In adult patients with cardiogenic shock, CCUS does not impact mortality, time to resolution of hemodynamic instability within 6 days, ICU and hospital LOS, nor mechanical ventilation or RRT duration. Notably, CCUS may hasten resolution of hemodynamic instability at 72 hours, but such evidence is limited by imprecision and indirectness.

**KEY POINTS**

**Question**: Does critical care ultrasonography (CCUS) improve outcomes in adult patients with cardiogenic shock?

**Findings**: CCUS has no effect on mortality, ICU and hospital length of stay, mechanical ventilation, or renal replacement therapy duration. CCUS may hasten hemodynamic instability resolution at 72 hours.

**Meanings**: CCUS may have a role in shortening the time to resolution of hemodynamic instability at 72 hours but the evidence is limited by imprecision and indirectness suggesting the need for additional focused inquiry.

Cardiogenic shock (CS), a syndrome where impaired cardiac output results in tissue hypoperfusion, frequently precipitates multiple organ failure and death (1). CS remains a leading cause of death after myocardial infarction (MI), complicates 5–10% of acute MI cases, and has a mortality rate exceeding 40% (2, 3). Initial management is complex and benefits from inotropic and frequently mechanical circulatory support (MCS). Critical care ultrasonography (CCUS) is a tool that enables early recognition of CS and may adaptively guide therapy.

CCUS is point-of-care ultrasonography performed and interpreted by an ICU clinician to enable rapid diagnosis, management, and invasive procedure guidance (4). CCUS is a diagnostic tool to assess cardiac function in real-time, guide-volume management, evaluate multiple organs, and support repeated assessment following therapeutic intervention. Cardiac CCUS, in particular, may help liberate patients from extracorporeal circulatory support during myocardial recovery (5). Despite its practical applications and routine use, evidence addressing patient-centered outcomes is limited and deficiencies have not been categorized to guide focused inquiry. We therefore performed a scoping review to examine data regarding the efficacy of CCUS vs. usual care without CCUS in adult CS patients.

## METHODS

We registered our scoping review protocol on PROSPERO (CRD42024524880) and used the Preferred Reporting Items for Systematic reviews and Meta-Analysis extension for Scoping Reviews statement to guide study design and reporting (**Appendix 1**, https://links.lww.com/CCX/B610). As a scoping review, ethics approval was not required.

### Databases Explored

We searched MEDLINE, Embase, and CENTRAL in conjunction with an expert librarian (from inception to February 2024). We also searched gray literature for ongoing trials on ClinicalTrials.gov and the World Health Organization International Clinical Trials Registry Platform.

### Study Selection

We included all randomized clinical trials (RCTs) and observational studies comparing CCUS to non-CCUS care in adult CS patients (≥ 18 yr old). We included studies that used any ultrasound examination as part of their intervention (inferior vena cava [IVC], transthoracic echocardiography [TTE], transesophageal echocardiography (TEE), lung ultrasound). Settings included the emergency department (ED), acute care unit, or ICU. We included studies that reported: short-term mortality (1 mo or less), time to escalation of support, time to mechanical support weaning, renal replacement therapy use, neurologic outcome at discharge, ICU length of stay (LOS), and adverse events (including transesophageal echocardiography complications). Studies deploying ultrasound examination(s) in their control arm were excluded.

### Data Extraction and Quality Assessment

Title and abstract screening were independently performed by three reviewers and in duplicate (S.S., K.L., J.B.). Full texts were subsequently reviewed for eligibility, with disagreements resolved by consensus (majority agreement). Data extraction occurred using pre-piloted forms.

We used the modified Cochrane Risk-of-Bias 2.0 tool for RCTs and the Risk of Bias in Nonrandomized Studies—of Interventions tool to assess observational studies. Disagreements were resolved by discussion and consensus with the help of a third reviewer as needed. The risk of bias for each domain was categorized as: 1) low risk of bias, 2) probable low risk of bias, 3) probable high risk of bias, and 4) high risk of bias where outcomes are likely to be significantly influenced (6).

## RESULTS

**Figure 1.**
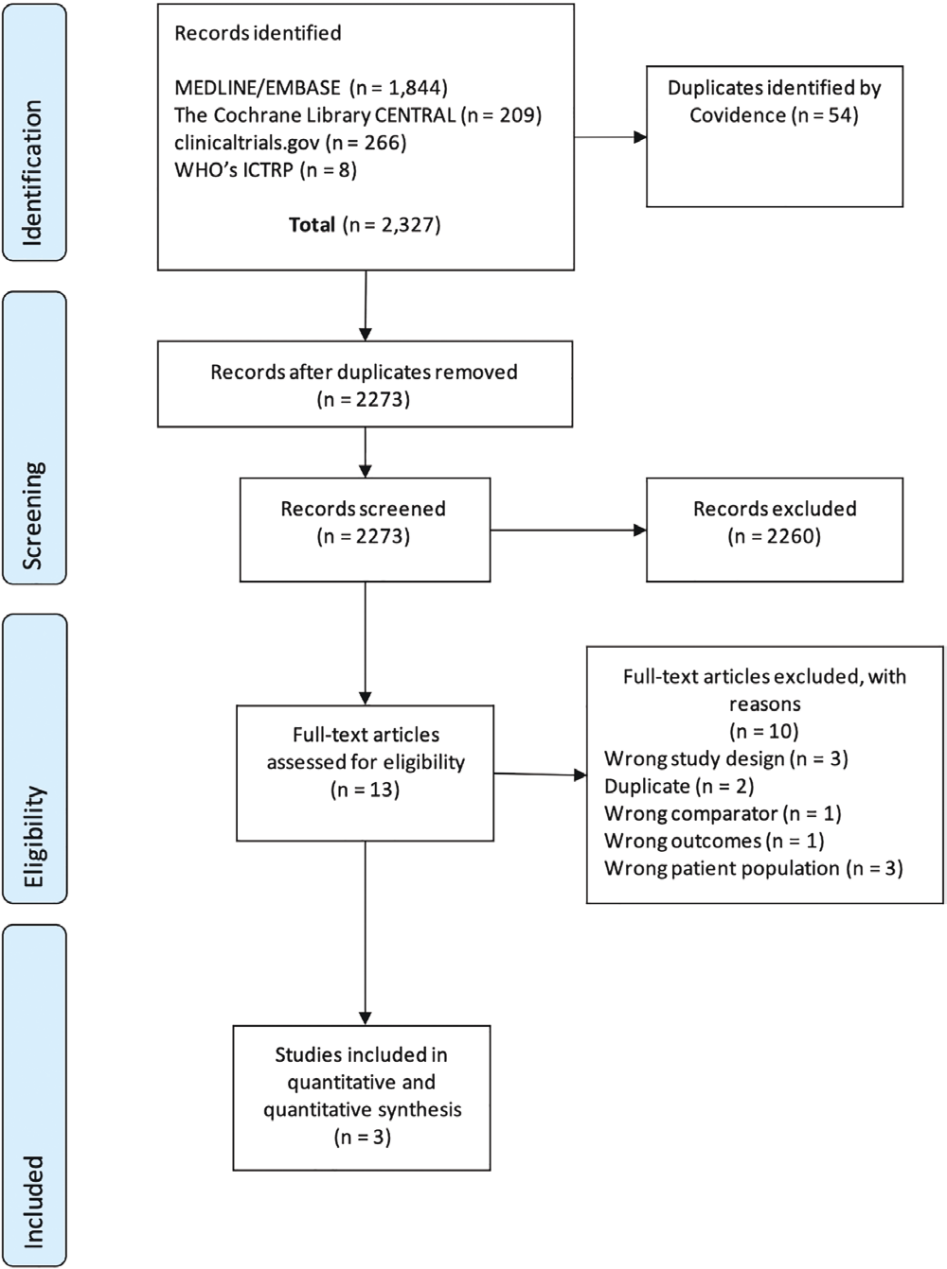
Study flow chart. ICTRP = International Clinical Trials Registry Platform, WHO = World Health Organization.

Database searches yielded 2329 citations (**Appendices 2–5**, https://links.lww.com/CCX/B610) that led to 13 full-text assessments. Selected studies included two RCTs (*n* = 573 patients) and one observational study (*n* = 30 patients; **Fig. 1**) (7–9). One RCT was a post hoc study evaluation (7). Trial characteristics are summarized in **Table 1**.

**TABLE 1.** Baseline Characteristics of Included Studies

Study Author and Year	Study Design and Setting	No. of Patients	Type of Ultrasound Performed	Control Arm	Ultrasound Operator	Inclusion Criteria	Exclusion Criteria
Atkinson et al (7) (2019)	Multicenter RCT in emergency department	CCUS: 138	Abdominal and cardiothoracic ultrasound as per the Rapid Ultrasound for Shock and Hypotension protocol	No Ultrasound	N/A	1) 19 yr old or older	1) Pregnancy known at presentation or discovered during initial screening
		Non-CCUS: 135				2) Presentation with a sustained initial systolic blood pressure less than 100 mm Hg, or a shock index > 1.0 (with systolic blood pressure < 120 mm Hg)	2) Necessity of cardiopulmonary resuscitation or other advanced cardiac life support interventions (e.g., defibrillation, emergency pacing, insertion of ventricular assist device) before screening or enrollment
							3) History of significant trauma in the past 24 hr
							4) Twelve-lead electrocardiogram diagnostic of acute myocardial infarction
							5) Evident clear mechanism or cause for the hypotension or shock
							6) Previously known diagnosis from another hospital (for transferred patients)
							7) Vagal episode (as cause of hypotension)
							8) Low blood pressure considered to be nonpathologic (normal variant or other)
Kanji et al (8) (2014)	Observational study in ICU	CCUS: 110	Transthoracic echocardiography (valve assessment and global left ventricle function)	Standard management	One of three ICU medical doctors with American College of Cardiology level II Echo certification	1) Patients referred to ICU with vasopressor dependent shock despite an IV fluid challenge achieving a central venous pressure of at least 8 mm Hg	Not reported
		Non-CCUS: 110					
Merz et al (9) (2019)	Single-center RCT in ICU	CCUS: 271	TEE	Standard Care without TEE	N/A	1) Patients > 18 yr old	1) Upper gastrointestinal tract pathology
		Non-CCUS: 274				2) Requiring mechanical ventilation	2) Cervical spine pathology
						3) Circulatory shock (mean arterial pressure < 60 mm Hg or < 80 mm Hg if chronic hypertension, for > 30 min despite fluid resuscitation or requiring vasopressors, and signs of hypoperfusion/organ dysfunction)	3) Severe coagulopathy (international normalized ratio > 3) precluding TEE
							4) ICU admission after planned surgery
							5) Patients on mechanical circulatory support

CCUS = critical care ultrasonography, N/A = not applicable, RCT = randomized clinical trial, TEE = transesophageal echocardiogram.

### Description of Included Studies

Of the three, one was a multicenter ED-based trial, while the other two were ICU anchored (7). All studies included patients with shock defined as: 1) sustained systolic blood pressure less than 100 mm Hg or a shock index greater than 1.0 (7); 2) vasopressor dependent shock despite an IV fluid challenge achieving a central venous pressure of greater than or equal to 8 mm Hg (8); and 3) mean arterial pressure less than 60 mm Hg or less than 80 mm Hg (in those with chronic hypertension) for greater than 30 minutes despite fluid resuscitation or requiring vasopressor therapy, accompanied by signs of hypoperfusion or organ dysfunction (9). All control arms used usual care without CCUS while intervention arms deployed CCUS.

The specific ultrasound examination performed varied across studies and included abdominal and cardiothoracic assessment via the Rapid Ultrasound for Shock and Hypotension (RUSH) protocol (7), TTE (8), and TEE (Table 1). Mean age was 66.5 years, mean Acute Physiology and Chronic Health Evaluation II (only two studies) was 24.7 (8, 9); 34.7% of study participants were female. The studies were conducted in Switzerland (9) and Canada (7, 8). Only one study specified the CCUS operator (medical doctor with American College of Cardiology level II Echo certification) (8). Study inclusion and exclusion criteria were variable but all included patients with shock, while two of the three excluded patients requiring MCS (7, 9). Study risk of bias is included as **Appendix 6** (https://links.lww.com/CCX/B610).

### Outcomes

The two RCTs comparing CCUS to usual care in CS patients found no mortality benefit (risk difference, –0.03; 95% CI, –0.1 to 0.05), nor reduction in overall time to resolution of hemodynamic instability within 6 days (subhazard ratio [SHR], 1.20; 95% CI, 0.98–1.46), ICU LOS (*p* = 0.87), hospital LOS (*p* = 0.91), or mechanical ventilation (*p* = 0.73), or renal replacement therapy duration (*p* = 0.68). However, the time to resolution of hemodynamic instability was shorter in the CCUS group at 72 hours (SHR, 1.26; 95% CI, 1.02–1.55). Importantly, the results were not stratified by cause of shock, although 205 of the 499 patients included had moderate to severely decreased left ventricular ejection fraction (LVEF). In the observational study, CCUS use was associated with decreased mortality (hazard ratio, 0.58; 95% CI, 0.34–0.99). However, this study only enrolled 30 patients and demonstrated imprecise results.

## DISCUSSION

This scoping review found that CCUS guided CS patient care exerts an unclear impact on mortality suggesting that additional inquiry is indicated—or that mortality is not an endpoint that is influenceable by CCUS-driven management, especially if there is multiple organ failure. Instead, other metrics may be more appropriate. For instance, while most endpoints were unimpacted by CCUS use, certain patients more rapidly gained hemodynamic stability by 72 hours. That this effect was not realized at 6 days, likely indicates slightly different patient populations despite presenting with CS. The purportedly narrow therapeutic window (i.e., 72 hr) seems applicable to CS patients requiring MCS who realized improved outcomes. Early MCS reduced all-cause hospital mortality perhaps by rapidly reversing shock (10, 11). While the sole study that noted this outcome did not stratify their results by shock type, 41.1% of patients had moderate to severely decreased LVEF establishing a reasonable parallel with CS (9). Because there are few studies with appropriate control and comparator groups—and a scarcity of high-quality data—CCUS’s utility and efficacy remain uncertain compared with non-CCUS guided management.

Nonetheless, CCUS is commonly used to diagnose and guide CS initial management including fluid resuscitation, fluid removal, or vasopressor initiation. It does not, however, directly impact the underlying cardiac pathophysiology. Furthermore, interoperator variability in image quality, evaluation completeness, and interpretation introduce substantial variability across trials, sites, and patients. Each of these elements may be enhanced by deploying real-time machine learning (ML)/augmented intelligence (AI) image interpretation to reduce variability and provide actionable decision-support. Such algorithms may be embedded in the medical device architecture or may be housed within a facility’s information technology infrastructure.

A ML/AI approach may help ensure high-quality CCUS imaging acquisition in critically ill patients that may be impeded by anasarca, body habitus, intra-abdominal hypertension, dressings, and therapeutic devices (12). Real-time image evaluation as well as technical guidance are realistic potentials for new technology incorporation into clinical practice. Alternatively, when appropriate transthoracic CCUS images cannot be obtained, TEE provides a high-fidelity solution, but requires technical expertise and is often accompanied by augmented sedation. Only one study examined TEE use and noted more rapid hemodynamic stability at 72 hours, perhaps indicating a unique patient population or advanced local expertise compared with studies focused on TTE.

Our evaluation is the first scoping review addressing CCUS for patient management and is strengthened by its protocol-based approach. Nonetheless, relevant limitations impair generalization based upon the small number of studies that reflect the requirement for an appropriate control arm. The large number of CCUS studies without a suitable control arm indicates broad utilization and sets the stage for focused inquiry, utilizing the areas of uncertainty noted within this scoping review. First, the exclusion of those with MCS precludes finding application to that specific population. Second, studies used multiple modalities and CCUS applications likely leading to heterogeneity and a lack of clear guidance. Third, the RUSH protocol is used for undifferentiated shock encompassing focused assessments of cardiac function, as well as the pleural space, IVC, aorta, and abdominal compartments; it is therefore not as specifically focused as cardiac CCUS and may be used in a more broad population that those presenting only with CS. Fourth, the skill set and training of the individual obtaining images is rarely reported, rendering direct comparison uncertain (8).

## CONCLUSIONS

In adult CS patients, CCUS is broadly used but studies that compare CCUS to no CCUS are few. Within the limited dataset, CCUS does not appear to beneficially impact most metrics of patient-centered outcomes. Importantly, CCUS obtained via TEE seems to shorten shock duration at 72 hours and suggests the need for additional inquiry into patient selection for TEE use as well as management. Underexplored domains include CCUS operator training, specific triggers for therapeutic decision-making, image adequacy, image interpretation fidelity, and interval change in serial assessments.

## ACKNOWLEDGMENTS

We thank Kaitryn Campbell, medical librarian and information specialist at St. Joseph’s Healthcare Hamilton, Faculty of Health Sciences, McMaster University, for her assistance in performing the comprehensive search of the databases.

## Supplementary Material
