# Supplementary material for: Critical Care Ultrasonography for Cardiogenic Shock: A Scoping Review
Source: Crit Care Explor. 2026 Mar 12;8(3):e1388. doi: 10.1097/CCE.0000000000001388 (PMC12987404; doi:10.1097/CCE.0000000000001388)
Supplement: Supplementary file 1 [file cc9-8-e1388-s001.pdf]

Supplementary Appendix

Supplement to: Sharif S, Wang K, Basmaji J, Ablordeppey E, Díaz-Gómez J, Lanspa M, Nikravan S, Lewis K. Critical Care Ultrasonography for Cardiogenic Shock: A Systematic Review.

Table of Contents

Appendix 1. PRISMA-ScR Checklist 3

Preferred Reporting Items for Systematic reviews and Meta-Analyses extension for Scoping Reviews (PRISMA-ScR) Checklist 3

Appendix 2. EMBASE, MEDLINE Search Strategy 5

Appendix 3. COCHRANE CENTRAL Search Strategy 9

Appendix 4. Clinical Trials.gov Search Strategy 12

Appendix 5. WHO International Clinical Trials Registry Platform (ICTRP) Search Strategy.....12

Appendix 6. Risk of Bias Tables 13

Appendix 1. PRISMA-ScR Checklist

Preferred Reporting Items for Systematic reviews and Meta-Analyses extension for Scoping Reviews (PRISMA-ScR) Checklist

SECTION	ITEM	PRISMA-ScR CHECKLIST ITEM	REPORTED ON PAGE #
**TITLE**			
Title	1	Identify the report as a scoping review.	1
**ABSTRACT**			
Structured summary	2	Provide a structured summary that includes (as applicable): background, objectives, eligibility criteria, sources of evidence, charting methods, results, and conclusions that relate to the review questions and objectives.	2
**INTRODUCTION**			
Rationale	3	Describe the rationale for the review in the context of what is already known. Explain why the review questions/objectives lend themselves to a scoping review approach.	4
Objectives	4	Provide an explicit statement of the questions and objectives being addressed with reference to their key elements (e.g., population or participants, concepts, and context) or other relevant key elements used to conceptualize the review questions and/or objectives.	4
**METHODS**			
Protocol and registration	5	Indicate whether a review protocol exists; state if and where it can be accessed (e.g., a Web address); and if available, provide registration information, including the registration number.	5
Eligibility criteria	6	Specify characteristics of the sources of evidence used as eligibility criteria (e.g., years considered, language, and publication status), and provide a rationale.	5-6
Information sources*	7	Describe all information sources in the search (e.g., databases with dates of coverage and contact with authors to identify additional sources), as well as the date the most recent search was executed.	5
Search	8	Present the full electronic search strategy for at least 1 database, including any limits used, such that it could be repeated.	5
Selection of sources of evidence†	9	State the process for selecting sources of evidence (i.e., screening and eligibility) included in the scoping review.	5-6
Data charting process‡	10	Describe the methods of charting data from the included sources of evidence (e.g., calibrated forms or forms that have been tested by the team before their use, and whether data charting was done independently or in duplicate) and any processes for obtaining and confirming data from investigators.	6
Data items	11	List and define all variables for which data were sought and any assumptions and simplifications made.	6
Critical appraisal of individual sources of evidence§	12	If done, provide a rationale for conducting a critical appraisal of included sources of evidence; describe	6-7

SECTION	ITEM	PRISMA-ScR CHECKLIST ITEM	REPORTED ON PAGE #
		the methods used and how this information was used in any data synthesis (if appropriate).	
Synthesis of results	13	Describe the methods of handling and summarizing the data that were charted.	7-9
**RESULTS**			
Selection of sources of evidence	14	Give numbers of sources of evidence screened, assessed for eligibility, and included in the review, with reasons for exclusions at each stage, ideally using a flow diagram.	14
Characteristics of sources of evidence	15	For each source of evidence, present characteristics for which data were charted and provide the citations.	14
Critical appraisal within sources of evidence	16	If done, present data on critical appraisal of included sources of evidence (see item 12).	14
Results of individual sources of evidence	17	For each included source of evidence, present the relevant data that were charted that relate to the review questions and objectives.	7-9
Synthesis of results	18	Summarize and/or present the charting results as they relate to the review questions and objectives.	7-9
**DISCUSSION**			
Summary of evidence	19	Summarize the main results (including an overview of concepts, themes, and types of evidence available), link to the review questions and objectives, and consider the relevance to key groups.	9-10
Limitations	20	Discuss the limitations of the scoping review process.	12
Conclusions	21	Provide a general interpretation of the results with respect to the review questions and objectives, as well as potential implications and/or next steps.	12
**FUNDING**			
Funding	22	Describe sources of funding for the included sources of evidence, as well as sources of funding for the scoping review. Describe the role of the funders of the scoping review.	13

JBI = Joanna Briggs Institute; PRISMA-ScR = Preferred Reporting Items for Systematic reviews and Meta-Analyses extension for Scoping Reviews.

* Where *sources of evidence* (see second footnote) are compiled from, such as bibliographic databases, social media platforms, and Web sites.

† A more inclusive/heterogeneous term used to account for the different types of evidence or data sources (e.g., quantitative and/or qualitative research, expert opinion, and policy documents) that may be eligible in a scoping review as opposed to only studies. This is not to be confused with *information sources* (see first footnote).

‡ The frameworks by Arksey and O'Malley (6) and Levac and colleagues (7) and the JBI guidance (4, 5) refer to the process of data extraction in a scoping review as data charting.

§ The process of systematically examining research evidence to assess its validity, results, and relevance before using it to inform a decision. This term is used for items 12 and 19 instead of "risk of bias" (which is more applicable to systematic reviews of interventions) to include and acknowledge the various sources of evidence that may be used in a scoping review (e.g., quantitative and/or qualitative research, expert opinion, and policy document).

From: Tricco AC, Lillie E, Zarin W, O'Brien KK, Colquhoun H, Levac D, et al. PRISMA Extension for Scoping Reviews (PRISMAScR): Checklist and Explanation. Ann Intern Med. 2018;169:467–473. doi: 10.7326/M18-0850.

Appendix 2. EMBASE, MEDLINE Search Strategy

#	Searches	Results
1	exp Cardiopulmonary Resuscitation/ use ppez	22860
2	exp *Resuscitation/ use oemezd	59337
3	exp Heart Arrest/ use ppez	57363
4	exp *Heart Arrest/ use oemezd	45472
5	(advanced cardiac life support or ACLS or asystole or ((cardiac or cardiopulmonary or cardio-pulmonary or cardiorespiratory or cardio-respiratory or cardiovascular or cardio-vascular or heart) adj (arrest* or life support* or resuscitat*)) or OHCA or pulseless electrical activity or "return of spontaneous circulation" or ROSC or sudden cardiac arrest? or sudden cardiac death).tw,kf,kw.	215516
6	or/1-5 [POPULATION Q1]	278962
7	Shock, Septic/ use ppez	25341
8	exp *Septic Shock/ use oemezd	24601
9	((endotoxic* or endotoxin or septic or sepsis or toxic) adj shock).tw,kf,kw.	95225
10	or/7-9 [POPULATION Q2]	106445
11	*Respiration Disorders/ use ppez	7600
12	exp *Respiratory Distress/ use oemezd	46658
13	Respiratory Distress Syndrome/ use ppez	25382
14	*Respiratory Tract Diseases/ use ppez	16814
15	*Respiratory Tract Disease/ use oemezd	21780
16	(((breathing or lung? or pulmonary) adj2 distress*) or (respirator* adj2 (deficien* or disease* or disorder* or disturbance? or distress* or dysfunction* or failure? or illness* or insufficien* or symptom*))).tw,kf,kw.	473533
17	(ARDS or ARDSS or acute respiratory distress syndrome? or adult respiratory distress or ((injury or shock) adj lung?) or ((posttraumatic or post-traumatic) adj (lung failure or pulmonary insufficiency))).tw,kf,kw.	86534
18	Acute Chest Syndrome/ use ppez	399
19	*Acute Chest Syndrome/ use oemezd	564
20	(acute chest adj syndrome?).tw,kf,kw.	4392
21	exp Dyspnea/ use ppez	25689
22	exp *Dyspnea/ use oemezd	16742
23	(breathlessness* or dyspnea? or dyspnoea* or ((difficult* or labored or laboured or short* or trouble*) adj2 (breath* or respiration))).tw,kf,kw.	238664
24	exp Hyperventilation/ use ppez	6758
25	exp *Hyperventilation/ use oemezd	4107
26	(hyperventilation? or hyper-ventilation? or overbreathing or over-breathing or respiratory alkalos#s).tw,kf,kw.	21713
27	exp Respiratory Insufficiency/ use ppez	69370
28	exp *Respiratory Failure/ use oemezd	27868
29	((respirator* or ventilator*) adj depression?).tw,kf,kw.	19547
30	((cardiopulmonary or cardio-pulmonary or lung? or respirator*) adj (arrest* or insufficien*)).tw,kf,kw.	36956
31	Tachypnea/ use ppez	323
32	*Tachypnea/ use oemezd	631
33	(polypnea? or polypnoea? or tachypnea? or tachypnoea? or tachydyspnoea?).tw,kf,kw.	15515
34	or/11-33	859194
35	Acute Disease/ use ppez	224584
36	*Acute Disease/ use oemezd	11857
37	(acute or acutely).tw,kf,kw.	3631146
38	exp Critical Care/ use ppez	67745
39	exp *Intensive Care/ use oemezd	288965

40	Critical Illness/ use ppez	40040
41	*Critical Illness/ use oemezd	14389
42	Critical Care Nursing/ use ppez	2630
43	*Intensive Care Nursing/ use oemezd	1233
44	exp Emergency Service, Hospital/ use ppez	101546
45	*Hospital Emergency Service/ use oemezd	2490
46	exp Emergency Medicine/ use ppez	15813
47	*Emergency Medicine/ use oemezd	30057
48	exp Intensive Care Units/ use ppez	108339
49	exp *Intensive Care Unit/ use oemezd	62413
50	Internal Medicine/ use ppez	19139
51	*Internal Medicine/ use oemezd	25417
52	(ICU$1 or MICU$1 or CICU$1 or CVICU$1 or CCU$1 or SICU$1 or POCCU$1 or ITU$1 or HDU$1).tw,kf,kw.	296327
53	(((acute* or critical*) adj2 (ill* or injur* or wound*)) or trauma*).tw,kf,kw.	1455448
54	((intensive* or critical* or neurointensive* or neuro-intensive* or neurocritical* or neuro-critical*) adj (care or therap* or treatment*)).tw,kf,kw.	618138
55	(high dependency or coronary care unit*).tw,kf,kw.	15848
56	exp Pneumonia/ use ppez	356237
57	exp *Pneumonia/ use oemezd	127488
58	(pneumon* or bronchopneumon* or pleuropneumon*).tw,kf,kw.	607292
59	exp Pneumothorax/ use ppez	18582
60	exp *Pneumothorax/ use oemezd	13654
61	(pneumothora* or pneumo-thora*).tw,kf,kw.	64665
62	exp Pleural Effusion/ use ppez	21705
63	exp *Pleural Effusion/ use oemezd	26460
64	pleural effusion*.tw,kf,kw.	77913
65	(Chylothorax/ or exp Empyema, Pleural/ or Hemopneumothorax/ or Hemothorax/ or Hydropneumothorax/ or Hydrothorax/) use ppez	12829
66	(*Chylothorax/ or *Pleural Empyema/ or *Hemopneumothorax/ or *Hematothorax/ or *Hydrothorax/) use oemezd	9664
67	(chylothor* or chylo-thor* or pleural empyem* or pyothora* or h?emopneumothora* or h?emo-pneumothora* or h?emothora* or h?emo-thora* or hydropneumothora* or hydro-pneumothora* or hydrothora* or hydro-thora*).tw,kf,kw.	33224
68	Pulmonary Edema/ use ppez	18018
69	*Lung Edema/ use oemezd	12568
70	((pulmonary or lung?) adj3 (edem* or oedem* or congestion)).tw,kf,kw.	66990
71	exp Heart Failure/ use ppez	151624
72	exp *Heart Failure/ use oemezd	266952
73	(heart adj (failure or insufficienc*)).tw,kf,kw.	603274
74	Pulmonary Embolism/ use ppez	43902
75	*Lung Embolism/ use oemezd	39273
76	((pulmonary or lung?) adj3 (embolism? or thromboembolism? or thrombo-embolism?)).tw,kf,kw.	127273
77	34 or ((or/35-55) and (or/56-76)) [POPULATION Q3]	1261764
78	exp Blood Volume/ use ppez	28171
79	exp *Blood Volume/ use oemezd	6104
80	exp Diuresis/ use ppez	21266
81	exp *Diuresis/ use oemezd	11482
82	exp Edema/ use ppez	46848
83	exp *Edema/ use oemezd	72099
84	Regional Blood Flow/ use ppez	66725
85	exp *Blood Flow/ use oemezd	100667

86	exp Water-Electrolyte Imbalance/ use ppez	67825
87	exp *Electrolyte Disturbance/ use oemezd	27504
88	bs.fs. use ppez	356680
89	(angioedema? or angio-edema? or diuresis or edema? or ((fluid? or volume?) adj2 (manag* or overload* or over-load* or status*)) or hypervolemi* or hyper-volemi* or lymphedema? or lymph-edema? or papilledema? or papill-edema? or (water-electrolyte? adj2 imbalance?)).mp.	746344
90	or/78-89 [POPULATION Q4]	1335758
91	Shock, Cardiogenic/ use ppez	11065
92	*Cardiogenic Shock/ use oemezd	11170
93	((cardiogen* or cardio-gen*) adj9 shock*).tw,kf,kw.	46216
94	(circulator* adj3 shock*).tw,kf,kw.	3830
95	coron* shock*.tw,kf,kw.	46
96	acute circulatory fail*.tw,kf,kw.	865
97	((undifferent* or undiagnos* or different or various or variety or type or types or kind or kinds or forms or states or etiologies) adj2 (shock* not heat shock*)).tw,kf,kw.	4963
98	or/91-97 [CARDIOGENIC SHOCK]	59285
99	(Myocardial Ischemia/ or Acute Coronary Syndrome/ or exp Myocardial Infarction/ or Percutaneous Coronary Intervention/ or Angioplasty, Balloon, Coronary/ or Myocardial Revascularization/ or Coronary Artery Bypass/) use ppez	328397
100	(*Heart Muscle Ischemia/ or exp *Acute Coronary Syndrome/ or exp *Heart Infarction/ or exp *Percutaneous Coronary Intervention/ or *Transluminal Coronary Angioplasty/ or *Heart Muscle Revascularization/ or *Coronary Artery Bypass Graft/) use oemezd	325295
101	(((card* or heart or myocard* or myo-card* or ST) adj3 (infarct* or isch?em*)) or (coronary intervent* or STEMI or PCI or angioplast* or post-isch* or postisch* or postinfarct* or postinfarct* or MI or AMI or myocard* revasc* or coronary artery bypass* or CABG or aortocoronary bypass* or aorto-coronary bypass* or coronary bypass*)).tw,kf,kw.	1181135
102	or/99-101 [MI]	1321544
103	(*Shock/ or shock.ti,kf,kw.) not (Electric Countershock/ or (septic or sepsis or bacter?em* or infect* or endotoxin* or heat shock or HSP* or anaphyl* or allergic or vasodilat* or osmotic or h?emorr* or bleeding or toxic or neurogen* or burn* or shock-wave or shock-resistant or hypovolemic or electroconvuls* or distributive shock*).ti,kf,kw.)	72182
104	(*Shock/ or shock.ti,kf,kw.) not (Cardioversion/ or (septic or sepsis or bacter?em* or infect* or endotoxin* or heat shock or HSP* or anaphyl* or allergic or vasodilat* or osmotic or h?emorr* or bleeding or toxic or neurogen* or burn* or shock-wave or shock-resistant or hypovolemic or electroconvuls* or distributive shock*).ti,kf,kw.)	72182
105	Cardiac Output, Low/ use ppez	5620
106	*Forward Heart Failure/ use oemezd	1472
107	((low* or diminish* or decreas* or declin* or reduc* or negligibl* or fall*) adj2 (card* or heart) adj output*).tw,kf,kw.	22645
108	((low* or diminish* or decreas* or declin* or reduc* or negligibl*) adj output*).tw,kf,kw.	5583
109	cardiac output low.tw,kf,kw.	172
110	(LCOS or COS or LCO).tw,kf,kw.	45646
111	((instab* or unstab*) adj1 h?emodyn*).tw,kf,kw.	30965
112	or/103-111 [SHOCK, LOW CO]	179084
113	102 and 112 [MI + CO]	23625
114	or/98,113 [POPULATION Q5]	69538
115	"Focused Assessment with Sonography for Trauma"/ use ppez	95
116	*"Focused Assessment with Sonography for Trauma"/ use oemezd	146
117	((focus* adj (abdomin* or assessment*) adj2 sonograph*) or FAST Exam* or eFAST Exam*).tw,kf,kw.	2511
118	(((app-based or bedside* or bed-side* or handheld or hand-held or handcarried or hand-carried or mobile or pocket* or point of care* or pointofcare* or poc or portable) adj2 (echo* or sonogr* or ultrason* or ultra-son* or ultrasound* or ultra-sound*)) or poc-lus or poc-us or pocus*).tw,kf,kw.	26366

119	((ultrasound* or ultra-sound* or ultrason* or ultra-son* or sonogr*) adj4 (lung* or pulmonary or heart or vena cava or chest or thorax or thoracic or transthoracic* or trans-thoracic* or cardio* or pleural or cardiac)).tw,kf,kw.	46382
120	or/115-119 [PoCUS 1]	69383
121	Point-of-Care Systems/ use ppez	16970
122	*"Point of Care System"/ use oemezd	1595
123	Point-of-Care Testing/ use ppez	4220
124	exp *"Point of Care Testing"/ use oemezd	8204
125	Mobile Applications/ use ppez	12173
126	exp *Mobile Application/ use oemezd	13825
127	(app-based or bedside* or bed-side* or handheld or hand-held or handcarried or hand-carried or ((mobile* or cordless or cellphone* or cell-phone* or phone*) adj2 app*) or pocket* or point of care* or pointofcare* or poc or portable).ti.	110665
128	((app-based or bedside* or bed-side* or handheld or hand-held or handcarried or hand-carried or ((mobile* or cordless or cellphone* or cell-phone* or phone*) adj2 app*) or pocket* or point of care* or pointofcare* or poc or portable) adj2 (assess* or comput* or diagnos* or system? or technolog* or test*)).tw,kf,kw.	65804
129	exp Critical Care/ use ppez	67745
130	exp *Intensive Care/ use oemezd	288965
131	Critical Illness/ use ppez	40040
132	*Critical Illness/ use oemezd	14389
133	Critical Care Nursing/ use ppez	2630
134	*Intensive Care Nursing/ use oemezd	1233
135	exp Emergency Service, Hospital/ use ppez	101546
136	*Hospital Emergency Service/ use oemezd	2490
137	exp Emergency Medicine/ use ppez	15813
138	*Emergency Medicine/ use oemezd	30057
139	exp Intensive Care Units/ use ppez	108339
140	exp *Intensive Care Unit/ use oemezd	62413
141	Internal Medicine/ use ppez	19139
142	*Internal Medicine/ use oemezd	25417
143	(ICU$1 or MICU$1 or CICU$1 or CVICU$1 or CCU$1 or SICU$1 or POCCU$1 or ITU$1 or HDU$1).tw,kf,kw.	296327
144	(((acute* or critical*) adj2 (ill* or injur* or wound*)) or trauma*).tw,kf,kw.	1455448
145	((intensive* or critical* or neurointensive* or neuro-intensive* or neurocritical* or neuro-critical*) adj (care or therap* or treatment*)).tw,kf,kw.	618138
146	(high dependency or coronary care unit*).tw,kf,kw.	15848
147	(emergency department? or internal medicine).tw,kf,kw.	419677
148	((intensivist* or internist? or physician? or clinician? or resident? or student?) adj6 (diagnos* or identif* or exam* or discriminat* or evaluat* or ultrasound* or sonogr* or ultrason* or echocardiogr* or LUS or LuCUS)).tw,kf,kw.	401383
149	or/121-148 [PoC/CC]	3202712
150	exp Ultrasonography/ use ppez	492781
151	exp *Echography/ use oemezd	254983
152	((medical* adj sonograph*) or ultrasonogram* or ultra-sonogram* or ultrasonograph* or ultra-sonograph* or ultrasonic* or ultra-sonic* or ultrasound* or ultra-sound*).tw,kf,kw.	1182368
153	(echocardiograph* or echo-cardiograph* or echocardiogram* or echo-cardiogram* or echogram* or echo-gram* or echograph* or echo-graph* or echotomograph* or echo-tomograph* or echotomogram* or echo-tomogram* or (surface? adj echo*) or VexUS).tw,kf,kw.	541838
154	or/150-153 [Ultrasound]	1891647
155	149 and 154 [PoCUS 2]	149959
156	120 or 155 [Intervention]	190897
157	(Case Reports/ or (case adj3 report?).ti.) use ppez	2454119

158	(exp Animals/ not (exp Animals/ and Humans/)) use ppez	5194605
159	((exp Child/ or exp Infant/) not ((exp Adult/ or exp Adolescent/) and (exp Child/ or exp Infant/))) use ppez	1484038
160	(Case Report/ or (case adj3 report?).ti.) use oemezd	3003430
161	(exp Animal/ not (exp Animal/ and exp Human/)) use oemezd	5209002
162	(exp Child/ not ((exp Adult/ or exp Adolescent/) and exp Child/)) use oemezd	1859673
163	or/157-162	18334009
164	(6 and 156) not 163 [Q1 Total]	5414
165	(202211* or 202212* or 2023* or 2024*).ed,dt. use ppez	2207610
166	(202211* or 202212* or 2023* or 2024*).dc,dd. use oemezd	2839776
167	or/165-166	5047386
168	164 and 167 [Q1 Total, update time period]	347
169	remove duplicates from 168 [Q1 Update Total, duplicates removed]	271
170	(10 and 156) not 163 [Q2 Total]	1603
171	170 and 167 [Q2 Total, update time period]	167
172	remove duplicates from 171 [Q2 Update Total, duplicates removed]	136
173	(77 and 156) not 163 [Q3 Total]	19556
174	173 and 167 [Q3 Total, update time period]	2181
175	remove duplicates from 174 [Q3 Update Total, duplicates removed]	1682
176	(90 and 156) not 163 [Q4 Total]	8586
177	176 and 167 [Q4 Total, update time period]	828
178	remove duplicates from 177 [Q4 Update Total, duplicates removed]	670
179	(114 and 156) not 163 [Q5 Total]	1749
180	179 and 167 [Q5 Total, update time period]	243
181	remove duplicates from 180 [Q5 Update Total, duplicates removed]	183

Appendix 3. COCHRANE CENTRAL Search Strategy

#	Searches	Results
1	exp Cardiopulmonary Resuscitation/	1481
2	exp Heart Arrest/	2917
3	(advanced cardiac life support or ACLS or asystole or ((cardiac or cardiopulmonary or cardio-pulmonary or cardiorespiratory or cardio-respiratory or cardiovascular or cardio-vascular or heart) adj (arrest* or life support* or resuscitat*)) or OHCA or pulseless electrical activity or "return of spontaneous circulation" or ROSC or sudden cardiac arrest? or sudden cardiac death).ti,ab,kw.	7747
4	or/1-3 [POPULATION Q1]	8600
5	Shock, Septic/	1269
6	((endotoxic* or endotoxin or septic or sepsis or toxic) adj shock).ti,ab,kw.	3673
7	or/5-6 [POPULATION Q2]	3805
8	Respiration Disorders/	470
9	Respiratory Distress Syndrome/	1941
10	Respiratory Tract Diseases/	588
11	(((breathing or lung? or pulmonary) adj2 distress*) or (respirator* adj2 (deficien* or disease* or disorder* or disturbance? or distress* or dysfunction* or failure? or illness* or insufficien* or symptom*))).ti,ab,kw.	22597
12	(ARDS or ARDSS or acute respiratory distress syndrome? or adult respiratory distress or ((injury or shock) adj lung?) or ((posttraumatic or post-traumatic) adj (lung failure or pulmonary insufficiency))).ti,ab,kw.	3880
13	Acute Chest Syndrome/	61
14	(acute chest adj syndrome?).ti,ab,kw.	224
15	exp Dyspnea/	2802

16	(breathlessness* or dyspnea? or dyspnoea* or ((difficult* or labored or laboured or short* or trouble*) adj2 (breath* or respiration))).ti,ab,kw.	15827
17	exp Hyperventilation/	326
18	(hyperventilation? or hyper-ventilation? or overbreathing or over-breathing or respiratory alkalos#s).ti,ab,kw.	1024
19	exp Respiratory Insufficiency/	3690
20	((respirator* or ventilator*) adj depression?).ti,ab,kw.	3802
21	((cardiopulmonary or cardio-pulmonary or lung? or respirator*) adj (arrest* or insufficien*)).ti,ab,kw.	1061
22	Tachypnea/	79
23	(polypnea? or polypnoea? or tachypnea? or tachypnoea? or tachydyspnoea?).ti,ab,kw.	756
24	or/8-23	45273
25	Acute Disease/	10726
26	(acute or acutely).ti,ab,kw.	166499
27	exp Critical Care/	2660
28	Critical Illness/	3254
29	Critical Care Nursing/	61
30	exp Emergency Service, Hospital/	3329
31	exp Emergency Medicine/	609
32	exp Intensive Care Units/	5257
33	Internal Medicine/	582
34	(ICU$1 or MICU$1 or CICU$1 or CVICU$1 or CCU$1 or SICU$1 or POCCU$1 or ITU$1 or HDU$1).ti,ab,kw.	19180
35	(((acute* or critical*) adj2 (ill* or injur* or wound*)) or trauma*).ti,ab,kw.	46476
36	((intensive* or critical* or neurointensive* or neuro-intensive* or neurocritical* or neuro-critical*) adj (care or therap* or treatment*)).ti,ab,kw.	32379
37	(high dependency or coronary care unit*).ti,ab,kw.	895
38	exp Pneumonia/	10373
39	(pneumon* or bronchopneumon* or pleuropneumon*).ti,ab,kw.	20692
40	exp Pneumothorax/	540
41	(pneumothora* or pneumo-thora*).ti,ab,kw.	2430
42	exp Pleural Effusion/	470
43	pleural effusion*.ti,ab,kw.	1870
44	Chylothorax/ or exp Empyema, Pleural/ or Hemopneumothorax/ or Hemothorax/ or Hydropneumothorax/ or Hydrothorax/	175
45	(chylothor* or chylo-thor* or pleural empyem* or pyothora* or h?emopneumothora* or h?emo-pneumothora* or h?emothora* or h?emo-thora* or hydropneumothora* or hydro-pneumothora* or hydrothora* or hydro-thora*).ti,ab,kw.	495
46	Pulmonary Edema/	310
47	((pulmonary or lung?) adj3 (edem* or oedem* or congestion)).ti,ab,kw.	2021
48	exp Heart Failure/	14592
49	(heart adj (failure or insufficienc*)).ti,ab,kw.	34272
50	Pulmonary Embolism/	1309
51	((pulmonary or lung?) adj3 (embolism? or thromboembolism? or thrombo-embolism?)).ti,ab,kw.	4915
52	24 or ((or/25-37) and (or/38-51)) [POPULATION Q3]	61660
53	exp Blood Volume/	1381
54	exp Diuresis/	1150
55	exp Edema/	2561
56	Regional Blood Flow/	3265
57	exp Water-Electrolyte Imbalance/	2748
58	(angioedema? or angio-edema? or diuresis or edema? or ((fluid? or volume?) adj2 (manag* or overload* or over-load* or status*)) or hypervolemi* or hyper-volemi* or lymphedema? or lymph-edema? or papilledema? or papill-edema? or (water-electrolyte? adj2 imbalance?)).af.	27495

59	or/53-58 [POPULATION Q4]	34490
60	Shock, Cardiogenic/	413
61	((cardiogen* or cardio-gen*) adj9 shock*).ti,ab,kw.	1517
62	(circulator* adj3 shock*).ti,ab,kw.	77
63	coron* shock*.ti,ab,kw.	0
64	acute circulatory fail*.ti,ab,kw.	40
65	((undifferent* or undiagnos* or different or various or variety or type or types or kind or kinds or forms or states or etiologies) adj2 (shock* not heat shock*)).ti,ab,kw.	137
66	or/60-65 [CARDIOGENIC SHOCK]	1800
67	Myocardial Ischemia/ or Acute Coronary Syndrome/ or exp Myocardial Infarction/ or Percutaneous Coronary Intervention/ or Angioplasty, Balloon, Coronary/ or Myocardial Revascularization/ or Coronary Artery Bypass/	29529
68	(((card* or heart or myocard* or myo-card* or ST) adj3 (infarct* or isch?em*)) or (coronary intervent* or STEMI or PCI or angioplast* or post-isch* or postisch* or postinfarct* or postinfarct* or MI or AMI or myocard* revasc* or coronary artery bypass* or CABG or aortocoronary bypass* or aorto-coronary bypass* or coronary bypass*)).ti,ab,kw.	71171
69	or/67-68 [MI]	75877
70	(*Shock/ or shock.ti,kf,kw.) not (Electric Countershock/ or (septic or sepsis or bacter?em* or infect* or endotoxin* or heat shock or HSP* or anaphyl* or allergic or vasodilat* or osmotic or h?emorr* or bleeding or toxic or neurogen* or burn* or shock-wave or shock-resistant or hypovolemic or electroconvuls* or distributive shock*).ti,kf,kw.)	1571
71	Cardiac Output, Low/	419
72	((low* or diminish* or decreas* or declin* or reduc* or negligibl* or fall*) adj2 (card* or heart) adj output*).ti,ab,kw.	965
73	((low* or diminish* or decreas* or declin* or reduc* or negligibl*) adj output*).ti,ab,kw.	175
74	cardiac output low.ti,ab,kw.	4
75	(LCOS or COS or LCO).ti,ab,kw.	542
76	((instab* or unstab*) adj1 h?emodyn*).ti,ab,kw.	1510
77	or/70-76 [SHOCK, LOW CO]	4943
78	69 and 77 [MI + CO]	1336
79	or/66,77 [POPULATION Q5]	5818
80	"Focused Assessment with Sonography for Trauma"/	2
81	((focus* adj (abdomin* or assessment*) adj2 sonograph*) or FAST Exam* or eFAST Exam*).ti,ab,kw.	70
82	(((app-based or bedside* or bed-side* or handheld or hand-held or handcarried or hand-carried or mobile or pocket* or point of care* or pointofcare* or poc or portable) adj2 (echo* or sonogr* or ultrason* or ultra-son* or ultrasound* or ultra-sound*)) or poc-lus or poc-us or pocus*).ti,ab,kw.	904
83	((ultrasound* or ultra-sound* or ultrason* or ultra-son* or sonogr*) adj4 (lung* or pulmonary or heart or vena cava or chest or thorax or thoracic or transthoracic* or trans-thoracic* or cardio* or pleural or cardiac)).ti,ab,kw.	2792
84	or/80-83 [PoCUS 1]	3563
85	Point-of-Care Systems/	578
86	Point-of-Care Testing/	185
87	Mobile Applications/	1622
88	(app-based or bedside* or bed-side* or handheld or hand-held or handcarried or hand-carried or ((mobile* or cordless or cellphone* or cell-phone* or phone*) adj2 app*) or pocket* or point of care* or pointofcare* or poc or portable).ti.	4997
89	((app-based or bedside* or bed-side* or handheld or hand-held or handcarried or hand-carried or ((mobile* or cordless or cellphone* or cell-phone* or phone*) adj2 app*) or pocket* or point of care* or pointofcare* or poc or portable) adj2 (assess* or comput* or diagnos* or system? or technolog* or test*)).ti,ab,kw.	2786
90	exp Critical Care/	2660
91	Critical Illness/	3254
92	Critical Care Nursing/	61

93	exp Emergency Service, Hospital/	3329
94	exp Emergency Medicine/	609
95	exp Intensive Care Units/	5257
96	Internal Medicine/	582
97	(ICU$1 or MICU$1 or CICU$1 or CVICU$1 or CCU$1 or SICU$1 or POCCU$1 or ITU$1 or HDU$1).ti,ab,kw.	19180
98	(((acute* or critical*) adj2 (ill* or injur* or wound*)) or trauma*).ti,ab,kw.	46476
99	((intensive* or critical* or neurointensive* or neuro-intensive* or neurocritical* or neuro-critical*) adj (care or therap* or treatment*)).ti,ab,kw.	32379
100	(high dependency or coronary care unit*).ti,ab,kw.	895
101	(emergency department? or internal medicine).ti,ab,kw.	15994
102	((intensivist* or internist? or physician? or clinician? or resident? or student?) adj6 (diagnos* or identif* or exam* or discriminat* or evaluat* or ultrasound* or sonogr* or ultrason* or echocardiogr* or LUS or LuCUS)).ti,ab,kw.	15591
103	or/85-102 [PoC/CC]	115590
104	exp Ultrasonography/	17616
105	((medical* adj sonograph*) or ultrasonogram* or ultra-sonogram* or ultrasonograph* or ultra-sonograph* or ultrasonic* or ultra-sonic* or ultrasound* or ultra-sound*).ti,ab,kw.	53071
106	(echocardiograph* or echo-cardiograph* or echocardiogram* or echo-cardiogram* or echogram* or echo-gram* or echograph* or echo-graph* or echotomograph* or echo-tomograph* or echotomogram* or echo-tomogram* or (surface? adj echo*) or VexUS).ti,ab,kw.	22145
107	or/104-106 [Ultrasound]	73780
108	103 and 107 [PoCUS 2]	6083
109	84 or 108 [Intervention]	8519
110	4 and 109 [Q1]	144
111	limit 110 to yr="2022 -Current" [Q1 Total, year limited]	25
112	7 and 109 [Q2]	129
113	limit 112 to yr="2022 -Current" [Q2 Total, year limited]	31
114	52 and 109 [Q3]	1356
115	limit 114 to yr="2022 -Current" [Q3 Total, year limited]	264
116	59 and 109 [Q4]	463
117	limit 116 to yr="2022 -Current" [Q4 Total, year limited]	83
118	79 and 109 [Q5]	209
119	limit 118 to yr="2022 -Current" [Q5 Total, year limited]	29

Appendix 4. Clinical Trials.gov Search Strategy

"Focused Assessment with Sonography for Trauma" OR FAST Exam OR eFAST Exam OR poc-lus OR poc-us OR pocus [intervention]
OR
bedside echography OR bedside ultrasound OR bedside sonography OR point of care echography OR point of care ultrasound OR point of care sonography OR portable echography OR portable ultrasound OR portable sonography [intervention]

=299 (266 results after duplicates removed)

Appendix 5. WHO International Clinical Trials Registry Platform (ICTRP) Search Strategy

"Focused Assessment with Sonography for Trauma" OR FAST Exam OR eFAST Exam OR poc-lus OR poc-us OR pocus [intervention]
OR
bedside echography OR bedside ultrasound OR bedside sonography OR point of care echography OR point of care ultrasound OR point of care sonography OR portable echography OR portable ultrasound OR portable sonography [intervention]

=19 results (8 after duplicates removed)

Appendix 6. Risk of Bias Tables

Table 1. Risk of Bias of Randomized Trials

Study Author and Year	Bias arising from the randomization process	Bias due to protocol deviations	Bias due to missing outcome data	Bias in measurement of the outcome	Bias in selection of the reported result	Overall ROB
**Atkinson 2019**	**Low**	**Low**	**Low**	**Probably Low**	**Probably Low**	**Probably Low**
**Merz 2019**	**Low**	**Low**	**Low**	**Low**	**Low**	**Low**

Table 2. Risk of Bias of Observational Studies

Study Author and Year	Bias due to confounding	Bias in selection of participants into the study	Bias in classification of Interventions	Bias due to deviations from intended interventions	Bias due to missing data/measurement of outcomes	Bias in selection of the reported result	Overall Risk of Bias
**Kanji 2014**	**Low**	**Serious**	**Low**	**Moderate**	**Low/Low**	**Low**	**Serious**
